# Clinical Features and Variations of Pain Expressions in 834 Burning Mouth Syndrome Patients With or Without Psychiatric Comorbidities

**DOI:** 10.7759/cureus.51139

**Published:** 2023-12-26

**Authors:** Chihiro Takao, Motoko Watanabe, Gayatri Nayanar, Trang Tu, Yojiro Umezaki, Miho Takenoshita, Haruhiko Motomura, Takahiko Nagamine, Akira Toyofuku

**Affiliations:** 1 Department of Psychosomatic Dentistry, Tokyo Medical and Dental University, Tokyo, JPN; 2 Department of Basic Dental Science, University of Medicine and Pharmacy at Ho Chi Minh City, Ho Chi Minh City, VNM; 3 Department of Geriatric Dentistry, Fukuoka Dental College, Fukuoka, JPN; 4 Department of Psychiatric Internal Medicine, Sunlight Brain Research Center, Yamaguchi, JPN

**Keywords:** oral psychosomatic disorders, psychosomatic dentistry, pain questionnaire, short-form mcgill pain questionnaire, pain catastrophizing scale, quality of the pain, psychiatric comorbidities, burning mouth syndrome

## Abstract

Introduction

Burning mouth syndrome (BMS) is characterized as chronic burning pain or unpleasant discomfort in the oral region without any corresponding clinical abnormalities. The aim of this study is to investigate the difference in clinical features and the variations of pain expressions between BMS patients with and without psychiatric comorbidities.

Methodology

The patients with BMS who first visited between April 2016 and March 2020 were involved and the clinical data including the presence of psychiatric comorbidities and scores of self-rating depression scale (SDS), pain catastrophizing scale (PCS), and pain quality from short-form McGill pain questionnaire (SF-MPQ) were collected retrospectively.

Results

In 834 patients with BMS (700 females, 63.9 ± 13.1 years old), 371 patients (44.5%) had psychiatric comorbidities. There was no significant between-group difference in demographic data. However, significantly higher scores were observed in SDS (p < 0.001) and PCS (p < 0.001) in the patients with psychiatric comorbidities. Moreover, the patients with psychiatric comorbidities showed significantly stronger pain intensity (p < 0.001) besides higher scores of each descriptor in SF-MPQ. In addition, they had chosen more descriptors in SF-MPQ (p < 0.001); furthermore, the number of selected pain descriptors showed a stronger correlation with PCS than with SDS regardless of the presence of psychiatric comorbidities.

Conclusion

BMS patients may complain of various pain expressions regardless of the psychiatric comorbidities; however, more severe complaints relating to high pain catastrophizing are more likely in patients with psychiatric comorbidities. These results suggested that underlying anxiety exacerbated the variety of pain expressions in BMS patients with psychiatric comorbidities.

## Introduction

Burning mouth syndrome (BMS), one of the most frequent syndromes in oral psychosomatic disorders [1,2], is characterized as chronic burning pain or unpleasant discomfort in the oral region without any corresponding clinical abnormalities [3]. BMS was found with a prevalence of 0.7% to 18.3% of the population [4,5], and predominantly in middle-aged women [4]. Oral symptoms are mainly observed on the tongue especially the tip of the tongue, and they are sometimes found on the palatal mucosa [6]. For detailed complaints, patients with BMS complain of oral symptoms with various expressions of not only burning but also aching, itching, stinging, numbness, and so on [4,7,8]. Besides these sensory aspects of pain, the emotional aspect of pain also has been frequently reported in patients with BMS [1]. However, until now, no previous studies have been conducted on affective pain in BMS patients in detail.

Psychiatric comorbidities in BMS were found in 22.2% to 71.6% [1,2,4,9], although the comorbidity of severe psychiatric disorders such as schizophrenia (0.8%) and bipolar disorders (1.6%) are rare [9]. The previous study revealed that depression and anxiety affect pain or unpleasant discomfort in BMS as well as chronic pain in the other body parts [10]. A negative aspect of pain perception itself also would induce anxiety and depression [11]. In addition, pain catastrophizing which is rumination and feeling hopeless about managing the pain also would affect acceptance of pain [5,11,12]. Moreover, BMS patients with psychiatric comorbidities complained of a stronger intensity of pain compared to the patients without psychiatric comorbidities [8]. The emotional and cognitive factors may affect not only pain severity but also the recognition and acceptance of pain in patients with BMS. The complex interplay between psychological factors and chronic pain remains unclear, yet comprehending and managing both aspects is crucial for the effective treatment of chronic pain, including BMS. When patients present with comorbid psychiatric disorders, the treatment response might vary compared to those without such comorbidities [2], therefore the treatment strategies for BMS patients, such as consultations regarding prescription details, differ clinically based on the presence or absence of comorbid psychiatric disorders.

Herein, we hypothesized that the BMS patients with psychiatric disorders show more severe and complicated oral symptoms with various expressions than the patients without psychiatric comorbidities. The aim of this study was to investigate the difference in clinical characteristics, the variations in pain expressions, and the factors associated with the resulting differences between BMS patients with and without psychiatric comorbidities.

## Materials and methods

Methods

Patients

A total 1,025 patients with BMS, who first visited Psychosomatic Dental Clinic in Tokyo Medical and Dental University, Hospital between April 2016 and March 2020, were retrospectively collected in this study. The inclusion criteria were the presence of burning sensation or pain in visibly healthy oral mucosa conforming to the criteria of the International Classification of Headache Disorders 3rd edition (ICHD-3) [3]. The diagnoses of BMS were confirmed by the dentists who were certificated by the Japanese society of Psychosomatic dentistry. As for the exclusion criteria, the patients who did not finish informed consent (n = 3), whose diagnosis overlapped with other oral psychosomatic disorders (n = 167) and whose clinical information were not completed (n = 21) were excluded. Finally, 834 patients with BMS were involved in the analysis in this study.

The clinical data including sex, age, the duration of illness, the location of pain symptoms, the presence of dental triggers, the presence of psychiatric comorbidities and scores of psychological questionnaires and pain scales were collected retrospectively from patients’ medical charts. As the same method in the previous studies [1,9,13,14], the psychiatric diagnosis and treatment histories were inquired for all patients with a psychiatric history. Psychiatric comorbidities were categorized with the Diagnostic and Statistical Manual of Mental Disorders (DSM-5) according to the referral letter from patients’ attending psychiatrists. All patients provided written informed consent. This study was approved by the Ethical Committee of Tokyo Medical and Dental University Dental Hospital (approval number: D2013-005).

Assessments

**Figure 1:**
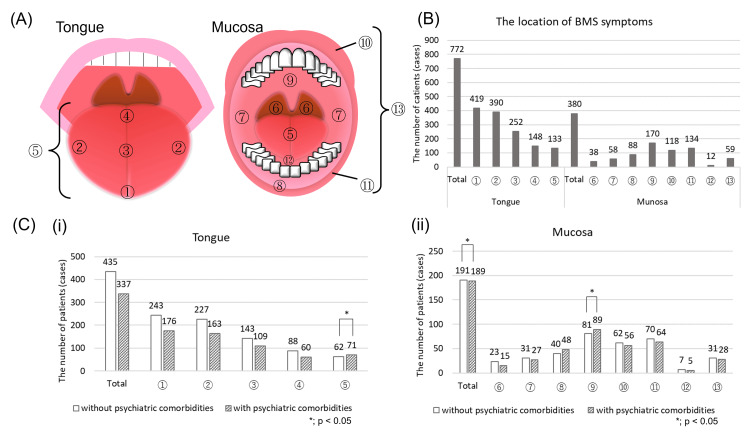
Distribution of pain locations (A) The atlas for oral pain distribution Pain localization was categorized into 14 areas as follows: ① tip of tongue, ② side of tongue, ③ dorsal tongue, ④ root of tongue, ⑤ tongue (unspecified), ⑥ throat, ⑦ buccal mucosa, ⑧ gingiva, ⑨ palate, ⑩ upper lip, ⑪ lower lip, ⑫ floor of mouth and ⑬ whole mouth (unspecified). (B) The BMS symptoms were mostly observed on tongue, especially the tip, followed by the mucosa of palatal area. (C) The differences in percentages of detailed pain distribution about tongue (i) and mucosa (ii) between the patients with and without psychiatric comorbidities. The pain on unspecific tongue, somewhere on the oral mucosa and palatal plate were detected significantly more in the patients with psychiatric comorbidities than those in the patients without psychiatric comorbidities by performing Peason's chi-square test.

Pain localization was categorized into 14 areas as follows: tip of tongue, side of tongue, dorsal tongue, root of tongue, whole tongue, throat, buccal mucosa, gingiva, palate, upper lip, lower lip, floor of the mouth, whole mouth and teeth (Figure 1A).

The laterality of the symptoms was also assessed according to patients’ complaints. The symptoms that were observed only on the tip of tongue, whole tongue or whole mouth were considered as bilateral symptoms.

The short-form McGill pain questionnaire (SF-MPQ) [15,16], which is commonly used to measure sensory, affective, and evaluative pain, is suitable for the assessment of various and complicated pain qualities in BMS patients for this study. In SF-MPQ, the sensory dimensions contain 11 descriptors as follows: throbbing, shooting, stabbing, sharp, cramping, gnawing, hot-burning, aching, heavy, tender, and splitting. The affective one contains the following four descriptors: tiring-exhausting, sickening, fearful, and punishing-cruel. These descriptors are evaluated with four grades (0 = none, 1 = mild, 2 = moderate, 3 = severe). SF-MPQ also includes the visual analog scale (VAS) and present pain intensity (PPI) to evaluate pain intensity. VAS assesses the severity of pain by using a 100mm slide line and where 0 presents no pain and 100 expresses worst pain they ever experienced. PPI measures pain on a scale of 0 to 5 where 0 = no pain, 1 = mild, 2 = discomforting, 3 = distressing, 4 = horrible, and 5 = excruciating.

Depressive states were assessed by using Zung's Self-Rating Depression Scale (SDS) [17] and the Japanese version of pain catastrophizing scale (PCS) [18] was used for measuring the tendency of rumination, magnify one's pain and helpless feeling to manage the pain.

For the use of Japanese version of SF-MPQ and PCS in this study, permission was obtained from the authors (Yamaguchi M and Matsuoka H, respectively), and SDS was bought from ©Sankyobo where all rights of SDS received.

Statistical analysis

The statistical software PASW for Windows version 17.0. (IBM Corp., Armonk, NY) was used for the analysis. Student t-test was performed for age, duration of the pain, self-rating depression scale, pain catastrophizing scale and VAS, the Peason's chi-square test was performed for sex and location of the pain and the Mann-Whitney U tests was for the score of Short-form McGill Pain Questionnaire except VAS to compare clinical characteristics between the BMS patients with and without psychiatric comorbidities. P-values of < 0.05 were considered statistical significance. Spearman's rank correlation coefficients were used to determine the significance of the correlation with Bonferroni correction. All data were described as mean ± standard deviation or median (first quantile. third quantile).

## Results

Demographic data and the results of psychological assessments

The 834 patients with BMS (700 females, 134 males, mean age; 63.9 ± 13.1 years old) were involved in this study (Table 1).

**Table 1:** Clinical characteristics in the patients with BMS with and without psychiatric comorbidities BMS: Burning mouth syndrome, SD: standard deviation All data were described as n (%) or mean ± standard deviation †: Pearson's chi-square test §: Student t-test *: p < 0.05 The range of total scores in Zung's self-rating depression scale is 20 to 80 with a cut-off score of 50, and that in pain catastrophizing scale is 0 to 52 with cut-off scores of 30.

Variables			Total (n=834)	Without psychiatric comorbidities (n = 463)	With psychiatric comorbidities (n = 371)	P-value	
Demographic data							
	Female/ male		700 / 134 (83.9% / 16.1%)	382 / 81 (82.5% / 17.5%)	318 / 53 (85.7% / 14.3%)	0.219	†
	Age, in years old		63.9±13.1	64.4±13.3	63.3±12.9	0.247	§
	Duration of BMS, in months		43.2±68.0	40.0±64.9	47.3±71.7	0.124	§
The presence of dental trigger			196 (23.5)	111 (24.9)	85 (22.9)	0.743	†
Comorbid psychiatric disorder							
	Absent		463 (55.5)	463 (100.0)	0 (0.0)	NA	†
	Present	Neurodevelopmental Disorders	2 (0.2)	0 (0)	2 (0.5)		
		Schizophrenia Spectrum and Other Psychotic Disorders	9 (1.1)		9 (2.4)		
		Bipolar and Related Disorders	9 (1.1)		9 (2.4)		
		Depressive Disorders	144 (17.3)		144 (38.8)		
		Anxiety Disorders	55 (6.6)		55 (14.8)		
		Obsessive-Compulsive and Related Disorders	5 (0.6)		5 (1.3)		
		Trauma-and Stressor-Related Disorders	5 (0.6)		5 (1.3)		
		Somatic Symptom and Related Disorders	52 (6.2)		52 (14.0)		
		Feeding and Eating Disorders	3 (0.4)		3 (0.8)		
		Sleep-Wake Disorders	55 (6.6)		55 (14.8)		
		Neurocognitive Disorders	9 (1.1)		9 (2.4)		
		Personality Disorders	1 (0.1)		1 (0.3)		
		Details unknown	80 (9.6)		80 (21.6)		
Total score of psychological assessment							
	Zung's self-rating depression scale		43.3±10.9	41.1±10.4	46.1±11.0	＜0.001	*§
	Pain catastrophizing scale		29.5±11.2	27.5±11.2	32.0±10.8	＜0.001	*§
Symptom's laterality							
		Unilateral	239 (28.7)	130 (28.1)	109 (29.4)	0.700	†
		Bilateral	595 (71.3)	333 (71.9)	262 (70.6)		

The mean duration of BMS was 43.2 ± 68.0 months. BMS symptoms were triggered by dental treatments in 196 patients (23.5%). The psychiatric comorbidities were observed in 371 patients (44.5%) in whom the most frequent diagnoses were depressive disorder (38.8%), followed by anxiety disorder (14.8%), sleep-wake disorders (14.8%) and somatic symptom and related disorders (14.0%). There was no significant difference between two groups in sex, age, the duration of illness and the presence of dental triggers; however, the scores of SDS and PCS for psychological assessments were significantly higher in the patients with psychiatric comorbidities than those in the patients without it (SDS: 41.1 ± 10.4 vs 46.1 ± 11.0, p < 0.001, PCS: 27.5 ± 11.2 vs 32.0 ± 10.8, p < 0.001, respectively).

For the pain distribution, the BMS symptoms were mostly observed on tongue (n = 772, 92.6%), especially the tip (n = 419, 50.2%) followed by the sides (n = 390, 46.8%), and the dorsal part (n = 252, 30.2%) as shown in Figure 1B. The pain in the other oral area except tongue was found in 380 patients (45.6%) mostly on the mucosa of palatal area (n = 170, 20.4%). Five hundred and ninety-five patients (71.3%) reported symptoms bilaterally whereas 239 patients (28.7%) reported unilateral symptoms. The pain on unspecific tongue (p = 0.028), somewhere on the oral mucosa (p = 0.006) and palatal plate (p = 0.024) were found significantly more in the patients with psychiatric comorbidities than those in the patients without; however, the laterality of symptoms showed no significant between-group differences (Figure 1C).

Pain characteristics

**Figure 2:**
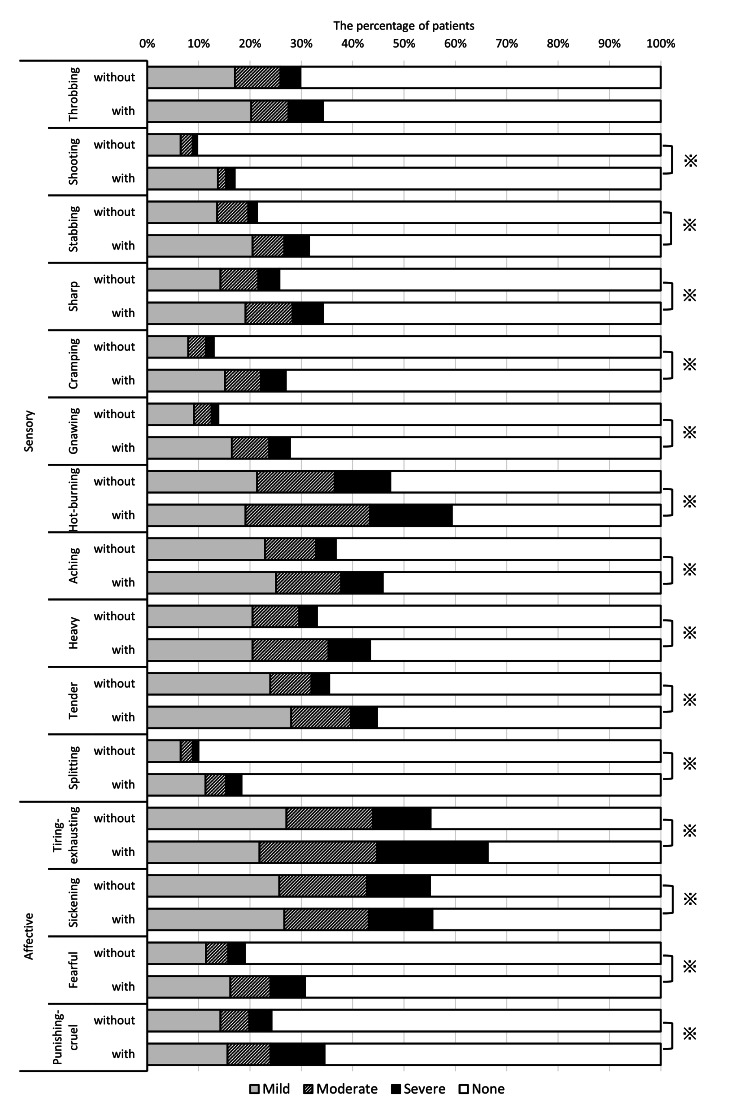
The scores of each descriptor in short-form McGill pain questionnaire All scores of each descriptor were significantly higher in the BMS patients with psychiatric comorbidities compared to the patients without it except throbbing, by performing Mann-Whitney U tests.

**Figure 3:**
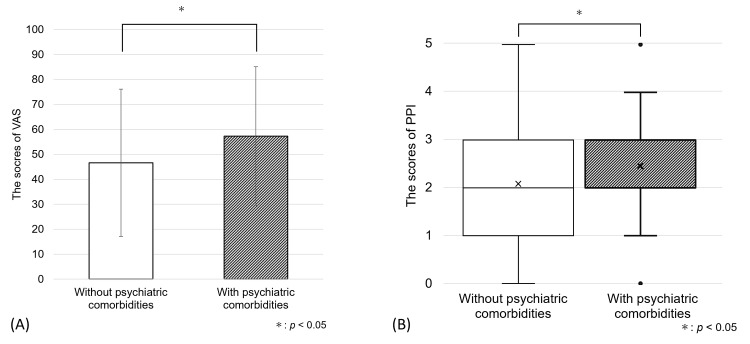
The scores of visual analogue scale (VAS, A) and present pain intensity (PPI, B) The average scores of VAS and PPI were significantly higher in the BMS patients with psychiatric comorbidities than those in the patients without psychiatric comorbidities. For the analysis of between-group differences, student t-test was performed for VAS, and Mann-Whitney U test was performed for PPI to analyze between-group differences respectively. The range of total scores in VAS is 0 to 100, and that in PPI is 0 to 5.

Various characteristics of pain was observed in this study. Regarding to sensory dimension, hot-burning (48.5%) was the most suffered pain, as were tiring-exhausting (61.9%) and sickening (50.7%) with regards to the affective dimension. Shooting and splitting types of pain were the least experienced. Not only all scores of each descriptor, except throbbing pain (Figure 2), but also the score of VAS and PPI were significantly higher in the patients with psychiatric comorbidities than those in the patients without psychiatric comorbidities (VAS: 46.6 ± 29.5 vs 57.3 ± 27.8, p < 0.001 PPI: 2.5 (1.3) vs 2.0 (2.3), p < 0.001, Figures 3A, 3B).

In addition, the total number of the selected descriptors in SF-MPQ were calculated since choosing many expressions for individual pain at the same time is quite interesting. The patients with psychiatric comorbidities had chosen more descriptors compared to the patients without psychiatric comorbidities (4.16 ± 3.54 vs 5.70 ± 4.32, p < 0.001). Although 72 patients had not selected any descriptors in SF-MPQ, they mainly complained of “like after get burned,” “tingling sensation,” and dysgeusia (Table 2).

**Table 2:** Detailed complaints in the patients who did not mark any descriptors in SF-MPQ All data were described as n: the number of patients who complained each detailed sensation.

Detailed complaints	Without psychiatric comorbidity (n = 54)	With psychiatric comorbidity (n = 18)
Tingling sensation	23	5
Like after get burned	20	5
Dysgeusia	11	5
Dryness	7	3
Feel unnatural	6	3
Pain	3	4
Rubbing teeth or denture	3	0
Rough sensation	2	0
Itchy	1	0
Swelling sensation	0	1
Sore	0	1

Correlations of the pain among the pain descriptors and psychological assessment

The correlations among the pain descriptors in SF-MPQ were shown in Table 3.

**Table 3:** Correlations among the descriptors in SF-MPQ in the patients with and without psychiatric comorbidities *: 0.2 < r ≤ 0.4 **: 0.4 < r ≤ 0.7 Bold letters indicate significant differences (p < 0.000222) analyzed by Spearman's rank correlation coefficients with Bonferroni correction.

Without psychiatric comorbidities																
		Throbbing	Shooting	Stabbing	Sharp	Cramping	Gnawing	Hot-burning	Aching	Heavy	Tender	Splitting	Tiring-exahausting	Sickening	Fearful	Punishing-cruel
	Throbbing															
	Shooting	0.389^*^														
	Stabbing	0.298^*^	0.458^**^													
	Sharp	0.309^*^	0.343^*^	0.532^**^												
	Cramping	0.269^*^	0.330^*^	0.249^*^	0.276^*^											
	Gnawing	0.238^*^	0.415^**^	0.384^*^	0.303^*^	0.530^**^										
	Hot-burning	0.058	0.113	0.261^*^	0.337^*^	0.174	0.176									
	Aching	0.313^*^	0.257^*^	0.256^*^	0.307^*^	0.287^*^	0.256^*^	0.155								
	Heavy	0.189^*^	0.249^*^	0.247^*^	0.234^*^	0.337^*^	0.291^*^	0.161	0.474^**^							
	Tender	0.106	0.131	0.226^*^	0.250^*^	0.154	0.234^*^	-0.010	0.192	0.236^*^						
	Splitting	0.307^*^	0.330^*^	0.349^*^	0.314^*^	0.318^*^	0.373^*^	0.193	0.260^*^	0.304^*^	0.227^*^					
	Tiring-exahausting	0.281^*^	0.290^*^	0.370^*^	0.427^**^	0.286^*^	0.305^*^	0.369^*^	0.354^*^	0.409^**^	0.163	0.289^*^				
	Sickening	0.235^*^	0.249^*^	0.287^*^	0.362^*^	0.293^*^	0.282^*^	0.265^*^	0.337^*^	0.416^**^	0.165	0.219^*^	0.624^**^			
	Fearful	0.234^*^	0.332^*^	0.300^*^	0.321^*^	0.291^*^	0.314^*^	0.360^*^	0.283^*^	0.337^*^	0.169	0.289^*^	0.532^**^	0.509^**^		
	Punishing-cruel	0.257^*^	0.349^*^	0.332^*^	0.323^*^	0.296^*^	0.266^*^	0.356^*^	0.251^*^	0.317^*^	0.118	0.381^*^	0.538^**^	0.452^**^	0.535^**^	
With psychiatric comorbidities																
	Throbbing															
	Shooting	0.454^**^														
	Stabbing	0.429^**^	0.503^**^													
	Sharp	0.388^*^	0.422^**^	0.627^**^												
	Cramping	0.391^*^	0.525^**^	0.481^**^	0.459^**^											
	Gnawing	0.465^**^	0.452^**^	0.508^**^	0.465^**^	0.632^**^										
	Hot-burning	0.219^*^	0.199	0.389^*^	0.373^*^	0.277^*^	0.236^*^									
	Aching	0.427^**^	0.381^*^	0.350^*^	0.405^**^	0.480^**^	0.454^**^	0.218^*^								
	Heavy	0.324^*^	0.343^*^	0.322^*^	0.382^*^	0.452^**^	0.417^**^	0.163	0.501^**^							
	Tender	0.245^*^	0.308^*^	0.252^*^	0.351^*^	0.301^*^	0.308^*^	0.053	0.271^*^	0.258^*^						
	Splitting	0.361^*^	0.461^**^	0.520^**^	0.423^**^	0.442^**^	0.506^**^	0.297^*^	0.327^*^	0.308^*^	0.339^*^					
	Tiring-exahausting	0.271^*^	0.208^*^	0.339^*^	0.398^*^	0.334^*^	0.302^*^	0.380^*^	0.374^*^	0.398^*^	0.192	0.239^*^				
	Sickening	0.372^*^	0.305^*^	0.340^*^	0.446^**^	0.380^*^	0.416^**^	0.293^*^	0.452^**^	0.461^**^	0.250^*^	0.301^*^	0.626^**^			
	Fearful	0.388^*^	0.338^*^	0.424^**^	0.394^*^	0.464^**^	0.446^**^	0.380^*^	0.470^**^	0.448^**^	0.246^*^	0.392^*^	0.549^**^	0.604^**^		
	Punishing-cruel	0.421^**^	0.284^*^	0.442^**^	0.467^**^	0.450^**^	0.451^**^	0.391^*^	0.446^**^	0.401^**^	0.245^*^	0.343^*^	0.591^**^	0.563^**^	0.661^**^	

Almost all descriptors correlated with each other regardless of the psychiatric comorbidities; moreover, many moderate correlations were observed in the patients with psychiatric comorbidities while weak correlations were observed in the patients without psychiatric comorbidities. Especially, the correlations among each descriptor in the affective dimension were moderately strong. For the correlations between the number of selected pain descriptors in SF-MPQ and the results of psychological assessments, a mild correlation with SDS (r = 0.222, p < 0.001; r = 0.302, p < 0.001), but moderate correlation with PCS (r = 0.404, p < 0.001; r = 0.550, p < 0.001) were observed in both patients with and without psychiatric comorbidities.

## Discussion

This study investigated the differences in clinical characteristics including the variation in the pain expressions between BMS patients with and without the comorbidity of psychiatric disorders. Our main findings were as follows: 1) The patients with psychiatric comorbidities showed more widespread and severe pain with significantly higher scores in each SF-MPQ descriptor compared to those without psychiatric comorbidities. 2) Almost all pain descriptors in SF-MPQ had correlations with each other and there were significantly higher correlations in the patients with psychiatric comorbidities; moreover, the number of selected descriptors showed a higher correlation in PCS than SDS regardless of the psychiatric comorbidities.

In this study, the patients with BMS were predominantly females (83.9%) in their middle age and with psychiatric comorbidities reported in about 44.5% [1,2,4,7,9]. There was no significant between-group difference in sex, age, or duration of illness. As with the distribution of the pain, 91.4% of the patients complained of symptoms on their tongue, especially on the tip of the tongue and side of the tongue, and 69.6% of the patients complained of symptoms present bilaterally. According to previous research, the tongue was the most frequent pain location regardless of the symptoms’ laterality [6,8]. Moreover, some researchers also revealed that the pain on the lip, palate, floor of the mouth, and gingiva was more frequent in the patients with psychiatric comorbidities compared to the patients without it [4,8]. In this study, more widespread pain was observed in the patients with psychiatric comorbidities without significant between-group differences in the symptoms’ laterality.

Concerning pain expressions, various expressions including hot-burning, aching, and tender were observed most frequently in SF-MPQ despite the presence of psychiatric comorbidities in this study. While burning and aching pain have been reported as the most frequent among BMS patients [8], other unpleasant and discomfortable sensations including dry mouth and dysgeusia were also reported [8,19]. In the present study, some patients without psychiatric comorbidities had not selected any descriptors in SF-MPQ; however, they complained of similar sensations through the medical interviews. For example, some patients had complained of a burning or tingling sensation but had not marked the descriptor “hot-burning” in SF-MPQ. They explained these symptoms were not exactly equal to pain but uncomfortable burning sensations. On the other hand, there are patients who had selected all 15 descriptors in SF-MPQ, especially those with psychiatric comorbidities. Their symptoms may apply to all the items of the SF-MPQ, but it is more likely that many items were selected because they struggle to express their “suffering” somehow more severely with various expressions rather than the pain sensation itself. These results may suggest that it is very difficult for patients to find the perfect expression for their actual pain. Thus, this indicates that the symptoms of BMS might be recognized not simply as pain but as something like tactual uncomfortable sensations accompanied by emotional aspects in some cases.

Cognitive and emotional factors have an enormous influence on pain perception, and a negative emotional state increases pain [11]. The number of items the patients selected in SF-MPQ had a positive correlation with PCS and SDS. Particularly, the score of PCS and the number of selected items were closely correlated. Pain catastrophizing is an important cognitive factor associated with pain perception or cognition and central sensitization [20]. High pain catastrophizing induces not only fear and anxiety for future pain but also a negative emotional response to actual pain [21,22]. Although chronic pain in BMS patients is affected by depression [23], these results suggested that catastrophizing reflecting anxiety might make the complaints of pain more variable and severe than depression.

Interestingly, although the main pain expressions of oral symptoms did not differ regardless of the presence of psychiatric comorbidities, more severe symptoms were found in all descriptors in the patients with psychiatric comorbidities. Moreover, high scores were observed in not only SDS but also PCS which had a moderate correlation with the number of selected pain descriptors in SF-MPQ. Furthermore, the patients with psychiatric comorbidities had a higher correlation among each pain descriptor in SF-MPQ than those without. The underlying anxiety may affect pain expressions and severity in more complicated mechanisms in patients with psychiatric comorbidities in speculation. Since psychological conditions such as anxiety change the activity of the central nervous system including pain-related regions [24], further investigations combined with brain imaging are needed.

There were several limitations in this study. First, since data were collected only at the first examination, the symptoms (pain, depression, and catastrophizing thinking) might have changed during the treatment course. A long-term follow-up study is thus required. Second, the patients were divided into groups based on the content of their referral letters from their attending psychiatrists who had seen them frequently, but not based on the assessment by specific psychiatrists. To assess psychiatric comorbidity which is one of the important indicators of the patient's characteristics, the diagnosis by attending psychiatrists would be clinically reliable to categorize patients into groups as the previous study showed [1,9,13,14]. Finally, all comorbid psychiatric disorders were categorized into the same group. As every psychiatric disorder with different conditions affected by pharmacological/non-pharmacological therapy might present different pain severities and qualities, categorizing them into separate groups would have helped garner more precise results. However, in this study since most of the psychiatric comorbidities were depressive disorders in the remission period and since severe disorders such as schizophrenia and bipolar disorders were few, it would not make any significance to analyze each psychiatric disorder.

## Conclusions

Conclusion

In conclusion, it can be inferred that BMS patients may complain of various pain expressions as per SF-MPQ regardless of the psychiatric comorbidities. However, more severe complaints relating to high pain catastrophizing are more likely in patients with psychiatric comorbidities. The study results have essentially suggested a role of underlying anxiety in the exacerbation of pain expression and severity in BMS patients with psychiatric comorbidities.
